# Aerodynamic Drag Analysis of 3-DOF Flex-Gimbal GyroWheel System in the Sense of Ground Test

**DOI:** 10.3390/s16122081

**Published:** 2016-12-07

**Authors:** Xin Huo, Sizhao Feng, Kangzhi Liu, Libin Wang, Weishan Chen

**Affiliations:** 1 Control and Simulation Center, Harbin Institute of Technology, Harbin 150080, China; fengsizhao1992@163.com (S.F.); lbwang_hit@126.com (L.W.); 2 Department of Electrical and Electronic Engineering, Chiba University, Chiba 263-8522, Japan; kzliu@faculty.chiba-u.jp; 3 School of Mechatronics Engineering, Harbin Institute of Technology, Harbin 150080, China; cws@hit.edu.cn

**Keywords:** GyroWheel, aerodynamic drag, dynamical modeling, numerical simulation, experimental verification, ground test

## Abstract

GyroWheel is an innovative device that combines the actuating capabilities of a control moment gyro with the rate sensing capabilities of a tuned rotor gyro by using a spinning flex-gimbal system. However, in the process of the ground test, the existence of aerodynamic disturbance is inevitable, which hinders the improvement of the specification performance and control accuracy. A vacuum tank test is a possible candidate but is sometimes unrealistic due to the substantial increase in costs and complexity involved. In this paper, the aerodynamic drag problem with respect to the 3-DOF flex-gimbal GyroWheel system is investigated by simulation analysis and experimental verification. Concretely, the angular momentum envelope property of the spinning rotor system is studied and its integral dynamical model is deduced based on the physical configuration of the GyroWheel system with an appropriately defined coordinate system. In the sequel, the fluid numerical model is established and the model geometries are checked with FLUENT software. According to the diversity and time-varying properties of the rotor motions in three-dimensions, the airflow field around the GyroWheel rotor is analyzed by simulation with respect to its varying angular velocity and tilt angle. The IPC-based experimental platform is introduced, and the properties of aerodynamic drag in the ground test condition are obtained through comparing the simulation with experimental results.

## 1. Introduction

Flexible gimbal systems are commonly used and of great significance in space technology and applications, ranging from flexible-joint space manipulators [1,2], space telescopes and solar panels [3] to other applied robots, mechatronics and integrated systems [4,5]. One of the most potential impacts of using flexible systems is to combine necessary subsystems where possible, into a lighter, cheaper, smaller, commercially available and proven system, while providing the reliability, accuracy and functionality required at the same time [6,7,8]. The study of flexible mechanisms has become a major development trend for spacecraft and satellites, which is also expected to continue in the future [9,10].

GyroWheel is one such system, which is an innovative attitude determination and control system (ADCS) device that provides both an angular momentum bias and control torques about three axes while also measuring angular rates about two axes perpendicular to the spin direction, i.e., this device is both an actuator and a sensor simultaneously [8,11,12,13]. The conception of GyroWheel was inspired by a Dynamically Tuned Gyroscope (DTG); however, it substantially departs from the classical DTG in structural parameters and operating principles due to its significantly larger rotor and tilt angle, as well as a time-varying angular velocity of the spinning rotor. All of these introduce design challenges, from both the perspective of implementation of torque outputs and measurement of attitude angular rates.

For GyroWheel to maintain a high orientation accuracy of the angular momentum vector and the operating smoothness of the spinning rotor, the prerequisite for achieving high precision measurement, should be guaranteed by well-designed ground tests and calibration. However, compared with the space environment, the Earth rotational rate, aerodynamic disturbance and gravitational acceleration inevitably affect the GyroWheel system in ground tests. Generally, the effect of Earth rotational rate and gravitational acceleration can be compensated for by utilizing multi-position calibration and rate testing [14,15,16]. However, the aerodynamic drag of GyroWheel system is difficult to be analyze due to the complexity of the airflow, the diversity and time-varying properties of the rotor motions. Although the problem of aerodynamic drag can be avoided by using a vacuum tank, the cost of the ground test will significantly increase considering that a temperature-controlled cabinet is indispensable due to the dependence of the GyroWheel parameters on the ambient temperature.

To the authors’ knowledge, little attention has been focused on the aerodynamic drag of the GyroWheel system in ground test conditions. Although the Canadian academics at Carleton University have partly studied the problem of ground test and calibration, the aerodynamic drag suffers from a lack of investigations [17]. Actually, few studies about flow field and gyros, such as DTG and liquid floated gyro, have been conducted. Ling had established the mathematical models of the disturbances caused by the inside gas of DTG in order to analyze the gas damping torque and gas dynamic pressure torque; however, the mathematical models lacked further validation [18]. Tang studied the flowing state of liquid medium for the high-speed rotor by FLUENT software and the MPIV (Micro Particle Image Velocimetry) technique [19]. The FLUENT software was used to simulate the closed flow field between the rotor and the stator by the Reynolds stress model of hydromechanics; then, the MPIV was used to observe the motion of flow field and to measure the speed of the flowing field. Li used FLUENT software to calculate the temperature field and inside flow field of the floater, and the results showed that the inside structure of the floater components has great influence on the medium flow field [20].

Motivated by the discussion above, the aerodynamic drag problem of the 3-DOF flex-gimbal GyroWheel system is investigated in this paper. With an appropriately defined coordinate system, the dynamical model of GyroWheel system is established from an energy point of view, and the airflow field around the GyroWheel rotor is simulated to analyze the properties of aerodynamic drag based on a commonly used CFD software FLUENT. In order to illustrate the reasonability and feasibility of numerical simulations, some experiments are developed based on the GyroWheel prototype platform, and then the properties of aerodynamic drag in the ground test condition are obtained through comparing the simulation and experimental results.

The rest of the paper is organized as follows. In Section 2, the physical structure of the GyroWheel system is described, including the property of angular momentum envelope. Additionally, the necessity of aerodynamic drag analysis is discussed according to the dynamical model of GyroWheel, which is established based on Lagrange’s method. The simulation model is established in the FLUENT environment in Section 3, where the mesh independence is checked and the aerodynamic drag property is mainly investigated with respect to the varying angular velocity of the spinning rotor, as well as its tilt angle. In Section 4, the experimental platform is introduced, and the simulation results are directly verified through comparison with the experimental results. Section 5 concludes this paper and outlines areas for future studies.

## 2. Problem Formulation

### 2.1. Description of 3-DOF GyroWheel System

#### 2.1.1. Physical Configuration and Coordinates

Inspired by Dynamically Tuned Gyro, the structure of GyroWheel system is based on DTG, but substantially departures from the classical DTG in physical parameters and working principle. It is never really tuned (as it is for a DTG), as its spinning velocity is constantly varying and it must be operated at relatively larger tilt angles. In this sense, it is essentially a form of double gimbal Control Moment Gyro (CMG), but using a spinning flex-gimbal system as opposed to the usual non-spinning motor driven gimbals that are typically used in CMG torque actuators. The torsional stiffness provided by the cross flexure pivots of the spinning flex-gimbal system enables the GyroWheel to be dynamically tuned, and the magnitude and orientation of the angular momentum vector can be reasonably changed simultaneously due to the innovative motions. Accordingly, the device functions as a two-axis rate sensing gyroscope at the same time as providing spacecraft control torques. By using the same size and power as a standard single axis momentum wheel of the same momentum class, it allows for significant reductions in the size, mass and power required, while still maintaining the same three-axis momentum steering capability.

**Figure 1:**
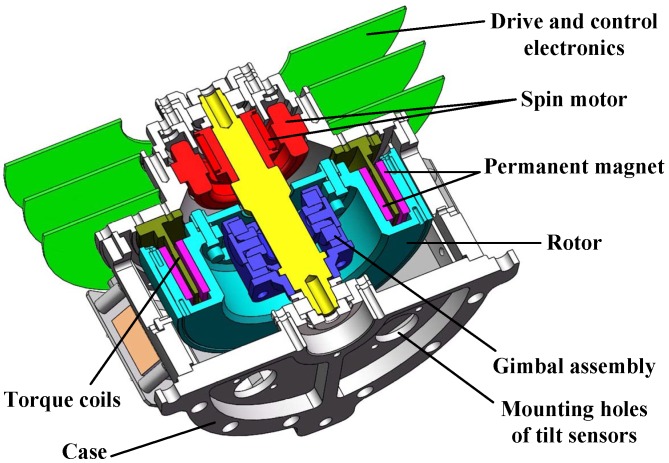
Cut-away view of the GyroWheel system.

Figure 1 shows a cutaway diagram that identifies the major components: case, spin motor, gimbal assembly, rotor, tilt sensors, permanent magnet and torque coils, drive and control electronics. A brushless DC motor spins the gimbal assembly and the rotor, and this motor is designed as part of the GyroWheel case. For a classical momentum or reaction wheel, the rotor would be mounted directly to the drive shaft. For GyroWheel, a specially designed flexure gimbal system is used instead. The gimbal assembly functions as a Hooke’s Joint, more commonly known as a universal joint, which allows the spinning wheel or rotor to tilt about both the axes perpendicular to the spin direction. The GyroWheel rotor is made of aluminum, with an annular-shaped gap on the underside. Permanent magnets are mounted to both interior surfaces of the rotor, and the torque coils are attached to the stationary case and are positioned between the inner and outer permanent magnet rings, which are used to create magnetic dipole moments. The orientation (or the tilt angle) of the rotor is measured by non-contact sensors installed associated to the upper surface of the case. A digital control system based on DSP is the kernel of the drive and control electronics, which is contained in the bottom half of the GyroWheel case.

**Figure 2:**
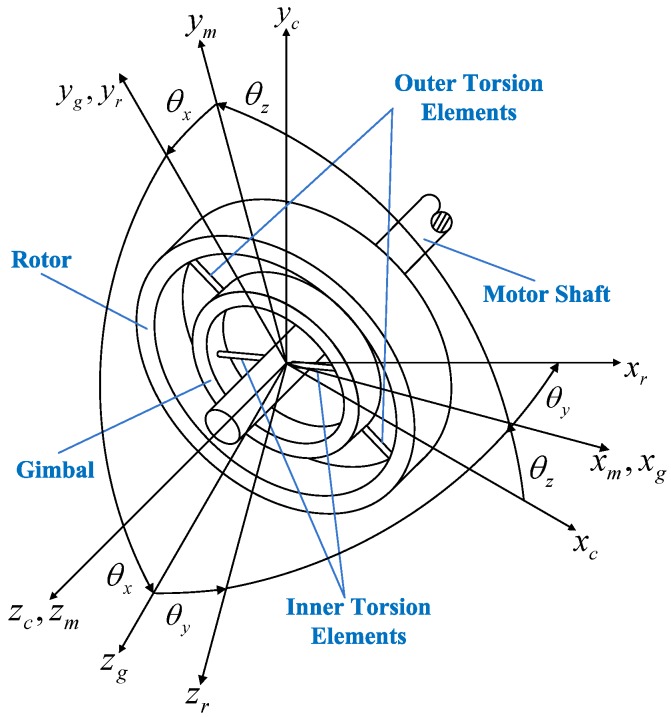
Coordinate frames with respect to the GyroWheel system.

According to the structure simplification, several coordinate frames can be defined with respect to the GyroWheel system, as shown in Figure 2. They are the case frame F${}_{c}$: O-x${}_{c}$y${}_{c}$z${}_{c}$, the motor frame F${}_{m}$: O-x${}_{m}$y${}_{m}$z${}_{m}$, the gimbal frame F${}_{g}$: O-x${}_{g}$y${}_{g}$z${}_{g}$ and the rotor frame F${}_{r}$: O-x${}_{r}$y${}_{r}$z${}_{r}$. The rotation angles $\theta_{x}$, $\theta_{y}$ and $\theta_{z}$, shown in Figure 2, are defined as generalized coordinates for the GyroWheel system and denote the motions about the z${}_{m}$-axis, x${}_{g}$-axis and y${}_{g}$-axis, respectively, which are used to describe the GyroWheel system within a spinning coordinate.

#### 2.1.2. Angular Momentum Envelope

Actually, the three-axis torque output is achieved based on the angular momentum exchange between the GyroWheel rotor and spacecraft when the GyroWheel is used as an actuator. In this section, the angular momentum envelope of the GyroWheel is investigated by considering both the time-varying angular velocity of the spinning wheel and its orientation in inertial space. According to the definitions above, the angular momentum H of the GyroWheel rotor can be simplified as follows:

(1) $$H=\left[\begin{array}{c} H_{x}\\ H_{y}\\ H_{z} \end{array}\right]=\left[\begin{array}{ccc} C_{\theta_{y}} & \;0 & \;S_{\theta_{y}}\\ S_{\theta_{x}}S_{\theta_{y}} & \;C_{\theta_{x}} & \;-S_{\theta_{x}}C_{\theta_{y}}\\ -C_{\theta_{x}}S_{\theta_{y}} & \;S_{\theta_{x}} & \;C_{\theta_{x}}C_{\theta_{y}} \end{array}\right]\left[\begin{array}{ccc} I_{rx} & \;0 & \;0\\ 0 & \;I_{ry} & \;0\\ 0 & \;0 & \;I_{rz} \end{array}\right]\left[\begin{array}{c} {\dot{\theta }}_{x}\\ {\dot{\theta }}_{y}\\ {\dot{\theta }}_{z} \end{array}\right]\approx \left[\begin{array}{c} I_{rz}{\dot{\theta }}_{z}S_{\theta_{y}}\\ -I_{rz}{\dot{\theta }}_{z}S_{\theta_{x}}C_{\theta_{y}}\\ I_{rz}{\dot{\theta }}_{z}C_{\theta_{x}}C_{\theta_{y}} \end{array}\right],$$

since ${\dot{\theta }}_{z}$ is much greater than ${\dot{\theta }}_{x}$ and ${\dot{\theta }}_{y}$. For the sake of simplicity, we use the notations $S_{\theta_{i}}$ and $C_{\theta_{i}}$ to replace $\sin\theta_{i}$ and $\cos\theta_{i}$ throughout the paper, where i=x,y,z.

It should be noted that $\theta_{x}$ and $\theta_{y}$ are time-varying with a given tilt angle, denoted by *ϕ*, for instance, where *ϕ* is finite, not necessarily equal to zero. In the case that the wheel tilts, a new variable of *β*, taking a value between 0 and 2π, is introduced to construct the relationship between $\theta_{x}$, $\theta_{y}$ and *ϕ* in the motor frame F${}_{m}$ as shown in the following:

(2) $$\left\{\begin{array}{c} \tan\theta_{x}=\tan\phi \cos\beta ,\\ \tan\theta_{y}=\tan\phi \sin\beta C_{\theta_{x}}, \end{array}\right.$$

where $\tan^{2}\phi =\tan^{2}\phi_{x}+\tan^{2}\phi_{y}$, $\phi_{x}$ and $\phi_{y}$ are the measured tilt angles of rotor by the tilt sensors in the case frame F${}_{c}$.

**Figure 3:**
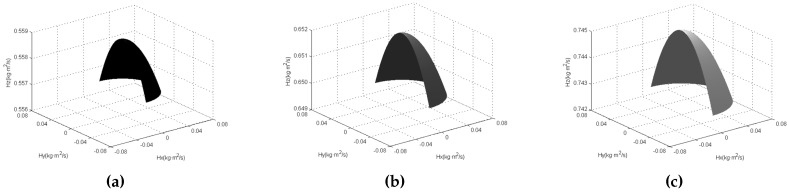
Angular momentum envelopes with different spinning angular velocities and limited tilt angles. (**a**) ${\dot{\theta }}_{z}=3000$ rpm; (**b**) ${\dot{\theta }}_{z}=3500$ rpm; (**c**) ${\dot{\theta }}_{z}=4000$ rpm.

**Figure 4:**
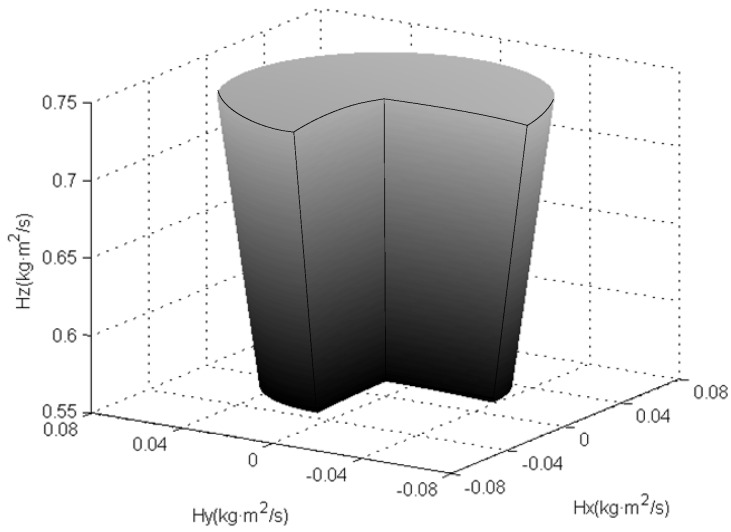
Cut-away view of the angular momentum envelope.

Substituting (2) into (1), and considering the physical parameters of the GyroWheel rotor listed in Table 1, the angular momentum workspace is obtained as shown in Figure 3 and Figure 4, respectively. In Figure 3, the angular momentum with constant spinning velocity and inconstant but finite tilt angle is displayed. Obviously, it is a part of a spherical surface whose radius is determined by the angular velocity of the rotor. Thus, the integral angular momentum workspace, including its cutaway view, is shown in Figure 4, which is a three-dimensional solid whose upper and lower surfaces are two spherical surfaces of different sizes. Hence, it is reasonable for the GyroWheel to provide exchangeable angular momentum in three-axis directions as an actuator like the variable speed double gimbal CMG [21].

### 2.2. Dynamical Modeling and Technical Challenge Formulation

Assuming the external rate of spacecraft is $\omega_{b}={\left[\omega_{bx}\;\omega_{by}\;\omega_{bz}\right]}^{T}$ in the case frame F${}_{c}$, the angular velocity of motor shaft in the motor frame F${}_{m}$ can be represented as

(3) $$\omega_{m}=\left[\begin{array}{c} 0\\ 0\\ {\dot{\theta }}_{z} \end{array}\right]+A_{c}^{m}\omega_{b}=\left[\begin{array}{c} \omega_{bx}C_{\theta_{z}}+\omega_{by}S_{\theta_{z}}\\ -\omega_{bx}S_{\theta_{z}}+\omega_{by}C_{\theta_{z}}\\ {\dot{\theta }}_{z}+\omega_{bz} \end{array}\right],$$

where $A_{c}^{m}$ denotes the transformation matrix from the case frame F${}_{c}$ to the motor frame F${}_{m}$.

Similarly, the angular velocity of gimbal in the gimbal frame F${}_{g}$ and the angular velocity of GyroWheel rotor in the rotor frame F${}_{r}$ can be obtained as follows:

(4) $$\omega_{g}=\left[\begin{array}{c} {\dot{\theta }}_{x}\\ 0\\ 0 \end{array}\right]+A_{m}^{g}\omega_{m}=\left[\begin{array}{c} {\dot{\theta }}_{x}+\omega_{bx}C_{\theta_{z}}+\omega_{by}S_{\theta_{z}}\\ -\omega_{bx}C_{\theta_{x}}S_{\theta_{z}}+\omega_{by}C_{\theta_{x}}C_{\theta_{z}}+\left({\dot{\theta }}_{z}+\omega_{bz}\right)S_{\theta_{x}}\\ \omega_{bx}S_{\theta_{x}}S_{\theta_{z}}-\omega_{by}S_{\theta_{x}}C_{\theta_{z}}+\left({\dot{\theta }}_{z}+\omega_{bz}\right)C_{\theta_{x}} \end{array}\right],$$

(5) $$\omega_{r}=\left[\begin{array}{c} 0\\ {\dot{\theta }}_{y}\\ 0 \end{array}\right]+A_{g}^{r}\omega_{g}=\left[\begin{array}{c} {\dot{\theta }}_{x}C_{\theta_{y}}-\dot{\theta_{z}}C_{\theta_{x}}S_{\theta_{y}}-\omega_{bz}C_{\theta_{x}}S_{\theta_{y}}+\left(C_{\theta_{y}}C_{\theta_{z}}-S_{\theta_{x}}S_{\theta_{y}}S_{\theta_{z}}\right)\omega_{bx}+\left(C_{\theta_{y}}S_{\theta_{z}}+S_{\theta_{x}}S_{\theta_{y}}C_{\theta_{z}}\right)\omega_{by}\\ {\dot{\theta }}_{z}S_{\theta_{x}}+{\dot{\theta }}_{y}+\omega_{bz}S_{\theta_{x}}-\omega_{bx}C_{\theta_{x}}S_{\theta_{z}}+\omega_{by}C_{\theta_{x}}C_{\theta_{z}}\\ {\dot{\theta }}_{x}S_{\theta_{y}}+\dot{\theta_{z}}C_{\theta_{x}}C_{\theta_{y}}+\omega_{bz}C_{\theta_{x}}C_{\theta_{y}}+\left(S_{\theta_{y}}C_{\theta_{z}}+S_{\theta_{x}}C_{\theta_{y}}S_{\theta_{z}}\right)\omega_{bx}+\left(S_{\theta_{y}}S_{\theta_{z}}-S_{\theta_{x}}C_{\theta_{y}}C_{\theta_{z}}\right)\omega_{by} \end{array}\right],$$

where $A_{m}^{g}$ and $A_{g}^{r}$ denote the transformation matrices from F${}_{m}$ to F${}_{g}$ and from F${}_{g}$ to F${}_{r}$, respectively.

In the sequel, the motor shaft, gimbal and rotor are regarded as rigid bodies, and notice that $\omega_{b}=0$ in the sense of the ground test. Hence, based on Lagrange’s method, the dynamical model of the GyroWheel system can be derived as follows (see the details in Appendix A for the derivation process):

(6) $$\begin{array}{c} \left[\begin{array}{ccc} I_{m1} & \;0 & \;I_{m2}\\ 0 & \;I_{ry} & \;I_{ry}S_{\theta_{x}}\\ I_{m2} & \;I_{ry}S_{\theta_{x}} & \;I_{m3} \end{array}\right]\left[\begin{array}{c} {\ddot{\theta }}_{x}\\ {\ddot{\theta }}_{y}\\ {\ddot{\theta }}_{z} \end{array}\right]+\left[\begin{array}{ccc} C_{x} & \;0 & \;0\\ 0 & \;C_{y} & \;0\\ 0 & \;0 & \;0 \end{array}\right]\left[\begin{array}{c} {\dot{\theta }}_{x}\\ {\dot{\theta }}_{y}\\ {\dot{\theta }}_{z} \end{array}\right]+\left[\begin{array}{ccc} K_{x} & \;0 & \;0\\ 0 & \;K_{y} & \;0\\ 0 & \;0 & \;0 \end{array}\right]\left[\begin{array}{c} \theta_{x}\\ \theta_{y}\\ \theta_{z} \end{array}\right]=\\ \left[\begin{array}{c} T_{gx}\\ T_{gy}\\ T_{gz} \end{array}\right]-\left[\begin{array}{ccc} C_{\theta_{y}} & \;0 & \;S_{\theta_{y}}\\ 0 & \;1 & \;0\\ -C_{\theta_{x}}S_{\theta_{y}} & \;-S_{\theta_{x}} & \;C_{\theta_{x}}C_{\theta_{y}} \end{array}\right]\left[\begin{array}{c} T_{dx}\\ T_{dy}\\ T_{dz} \end{array}\right]+\left[\begin{array}{c} F_{nl1}\\ F_{nl2}\\ F_{nl3} \end{array}\right], \end{array}$$

where $\theta_{x}$, $\theta_{y}$, $\theta_{z}$ are chosen to be generalized coordinates, $C_{x}$ and $C_{y}$ denote the damping coefficients of the inner and outer torsion elements, $K_{x}$ and $K_{y}$ denote the torsional rigidity of the inner and outer torsion elements, $T_{g}={\left[T_{gx}\;T_{gy}\;T_{gz}\right]}^{T}$ denotes the nonconservative generalized control torques applied to the GyroWheel, $T_{d}={\left[T_{dx}\;T_{dy}\;T_{dz}\right]}^{T}$ denotes the disturbance torques caused by aerodynamic drag which can be divided into the transverse disturbance torques $T_{dx}$, $T_{dy}$, and the spin disturbance torque $T_{dz}$, $F_{nl}={\left[F_{nl1}\;F_{nl2}\;F_{nl3}\right]}^{T}$ denotes the nonlinear torque term (see the details in Appendix B for the concrete expression).

In practice, the rotation angle of motor shaft $\theta_{z}$ can be measured by the Hall sensors; however, $\theta_{x}$ and $\theta_{y}$, defined in the moving coordinate system F${}_{r}$, is unmeasurable. According to the configuration of the GyroWheel system, the tilt angles $\phi_{x}$ and $\phi_{y}$, which are relative to the case, can be measured by the tilt sensors, and the relationship between ($\theta_{x}$ , $\theta_{y}$) and ($\phi_{x}$, $\phi_{y}$) is formulated as follows:

(7) $$\left[\begin{array}{c} \tan\phi_{x}\\ \tan\phi_{y} \end{array}\right]=\left[\begin{array}{c} \frac{S_{\theta_{x}}C_{\theta_{y}}C_{\theta_{z}}-S_{\theta_{y}}S_{\theta_{z}}}{C_{\theta_{x}}C_{\theta_{y}}}\\ \frac{S_{\theta_{x}}C_{\theta_{y}}S_{\theta_{z}}+S_{\theta_{y}}C_{\theta_{z}}}{C_{\theta_{x}}C_{\theta_{y}}} \end{array}\right].$$

Similarly, the nonconservative generalized control torques $T_{gx}$ and $T_{gy}$ are also unmeasurable, but the control torques $T_{cx}$ and $T_{cy}$ can be obtained by measuring the currents of the torque coils, where the relationship between them is given as follows:

(8) $$\left[\begin{array}{c} T_{gx}\\ T_{gy} \end{array}\right]=\left[\begin{array}{cc} C_{\theta_{z}} & \;S_{\theta_{z}}\\ -S_{\theta_{z}}C_{\theta_{x}} & \;C_{\theta_{z}}C_{\theta_{x}} \end{array}\right]\left[\begin{array}{c} T_{cx}\\ T_{cy} \end{array}\right].$$

Generally, the ground test of the GyroWheel system consists of a specification performance test and ground calibration. The objective of the specification performance test is to guarantee a high accuracy of orientation and magnitude control associated with the angular momentum vector, which is arranged in a lab environment. Meanwhile, the behaviours of the GyroWheel system can be better understood during the process of the ground test. According to the Lagrangian dynamical model of the GyroWheel system, the system itself is a non-linear, strong-coupled and time-varying parameter plant, plus multi-source mixed-frequency disturbances due to the flex-gimbal structure and innovative patterns of motion [8,11,12,13,17]. Compared with the space environment, the Earth rotational rate, aerodynamic disturbance and gravitational acceleration inevitably act on the GyroWheel system in the ground test. There are some schemes that can be used to compensate for errors caused by the Earth rotational rate and gravitational acceleration, such as utilizing dynamical balancing and calibration approaches. However, the aerodynamic drag, one of the main sources of interference in the lab environment, should never be overlooked because of the complexity of the airflow, the diversity and time-varying properties of the rotor motions.

On the other hand, the ground calibration, including multi-position calibration and rate testing, is usually implemented on a precision turntable, two-axis or three-axis, with a temperature-controlled cabinet. This is because the parameters of the mechanical gyro are always dependent on the ambient temperature, such as the moment coefficients of the torque coils and the damping coefficients of the inner/outer torsion elements. It is optimal to totally simulate the space environment during the calibration process. Unfortunately, this work is costly and arduous technically if an extra vacuum tank is considered. As a result, the aerodynamic drag analysis is beneficial to achieving a better understanding of the control properties for this plant, which, in turn, provides the possibility to improve the control performance of the non-linear GyroWheel system and further heighten the accuracy of ground calibration, and, simultaneously, the cost of ground test can be reduced significantly.

## 3. Numerical Simulations

### 3.1. Numerical Modeling in FLUENT

At present, FLUENT is one of the most popular and suitable pieces of computational fluid dynamics (CFD) software that is extensively applied in engineering fields and science studies [22,23,24,25]. It can be used to simulate and calculate complex flowing problems stably and efficiently including the laminar and turbulent, steady and transient flows, compressible and incompressible flows, the convective heat transfer and other issues. FLUENT can achieve the optimal convergence speed and solution precision due to the various solution methods, multi-grid acceleration techniques, flexible unstructured grids, self-adaptive grids method and comprehensive physical model.

#### 3.1.1. Model Geometries

**Figure 5:**
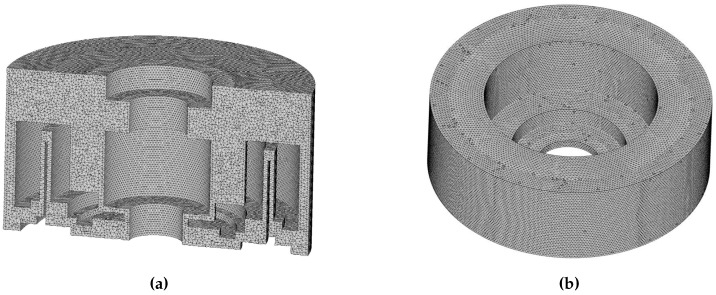
Schematic of the tetrahedral mesh with a number of 1987570. (**a**) Body mesh around the GyroWheel system; (**b**) Surface mesh of the GyroWheel rotor.

As shown in Figure 5, a 3D model is developed to simulate the inner airflow field around the GyroWheel rotor and the surface of the moving parts, where the boundary type is defined as `Wall’. Both of them are built and meshed in the pre-processing module of FLUENT software-GAMBIT under reasonable assumptions where the systematic and environmental parameters are listed in Table 2. Notice that the tilt angle in our simulation examples changes from $-5^{\circ }$ to +5${}^{\circ }$. Regarding the complexity of the inner airflow structure, the tetrahedron mesh is used to partition the whole computational domain, representing complex geometries [26].

**Figure 6:**
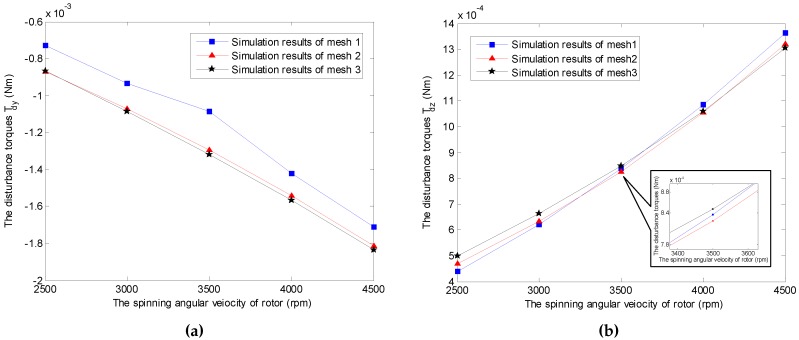
Mesh independence tests under $\phi_{x}=0^{\circ }$ and $\phi_{y}=0^{\circ }$ (numbers of mesh1, mesh2 and mesh3 are 271,399, 1,217,196 and 1,987,570, respectively). (**a**) Simulation results of $T_{dy}$; (**b**) Simulation results of $T_{dz}$.

The mesh density is crucial to the numerical simulation from the viewpoints of computation accuracy and time consumption. It is necessary to meet a balance between the simulation requirements, e.g., the accuracy, and the consumption of the CPU time. Furthermore, the number of meshes should be sufficient in order to obtain mesh independent results [27,28]. In our simulation, the independence of computing mesh is verified by three cases considering the whole working space of the GyroWheel rotor, as shown in Figure 6, where the numbers of mesh1, mesh2 and mesh3 are 271,399, 1,217,196 and 1,987,570, respectively. Without loss of generality, the computation results of the transverse aerodynamic torque $T_{dy}$ are selected for comparison. Obviously, the mesh independence is guaranteed when the mesh number is more than 1,217,196, where the results are convergent with respect to the increase of the mesh number. Therefore, the number of meshes used throughout this paper is chosen as 1,987,570 in the following simulations.

#### 3.1.2. Governing Equation

**Figure 7:**
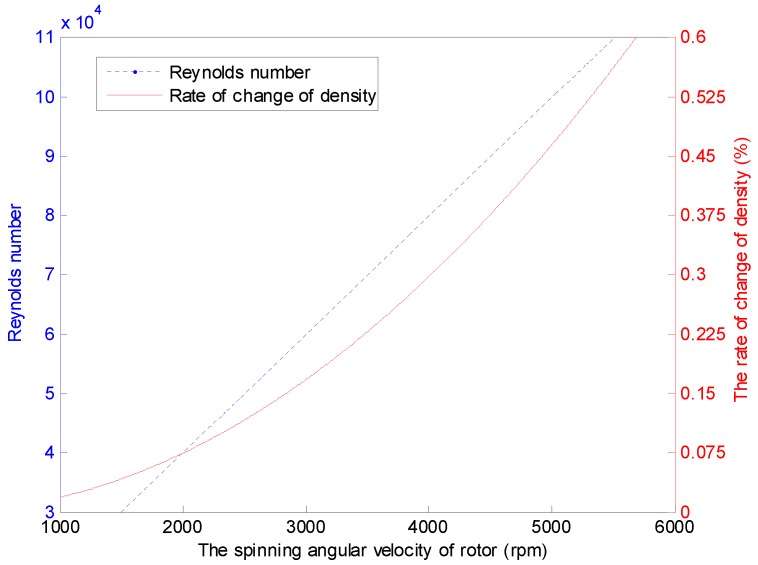
Reynolds number and relative density of the airflow of the GyroWheel under $\phi_{x}=0^{\circ }$, $\phi_{y}=0^{\circ }$ and other conditions listed in Table 2.

Similarly to the flow around a rotating disk in a cylindrical casing and the flow around the impeller in a tank, the state of the inner airflow motion around the GyroWheel rotor can be described by the Reynolds number, which is a dimensionless number as shown in (9), where, in fact, the Reynolds number is represented by the ratio of the non-viscous force to the viscous force [29,30]. Meanwhile, the change rate of the air density can be represented by (10), where the spinning angular velocity of the GyroWheel rotor varies under isothermal condition. As is indicated in Figure 7, the Reynolds number of airflow motion around the GyroWheel rotor is extremely large, but the relative density change with respect to the angular velocity of the spinning rotor is relatively small, which means that the airflow is turbulent and incompressible:

(9) $$Re=\frac{\rho R_{r}^{2}\omega_{rz}}{\mu },$$

(10) $$\frac{d\rho }{\rho }=\frac{\gamma }{2}M^{2},$$

where $\omega_{rz}$ denotes the angular velocity of the rotor, the specific heat ratio *γ* = 1.4, and *M* denotes Mach number of airflow, which is typically less than 0.3.

Regarding incompressible flows, the conservation equations of mass and momentum are used to describe the transient fluid flow in FLUENT are expressed as follows:

(11) $$\frac{\partial \rho }{\partial t}+\frac{\partial (\rho u_{j})}{\partial x_{j}}=0,$$

(12) $$\frac{\partial (\rho u_{i})}{\partial t}+\frac{\partial (\rho u_{i}u_{j})}{\partial x_{j}}=\partial \left[\mu (\frac{\partial u_{i}}{x_{j}}+\frac{\partial u_{j}}{x_{i}})\right]/\partial x_{j}-\frac{\partial (\rho u_{i})}{\partial x_{i}}+S_{i},$$

where $x_{i}$ and $x_{j}$ (i,j=1,2,3) denote the coordinate components, $u_{i}$ and $u_{j}$ denote the velocities, and $S_{i}$ denotes the source term.

In order to obtain relatively accurate results with less computer memory and CPU time, the k−ε standard turbulence model, due to its fast convergence speed, robust performance and wide application in engineering [31,32], is chosen to simulate the turbulent flow here:

(13a) $$\frac{\partial (\rho k)}{\partial t}+\frac{\partial (\rho ku_{j})}{\partial x_{j}}=\frac{\partial }{\partial x_{j}}\left[\left(\mu +\frac{\mu_{t}}{\sigma_{k}}\right)\frac{\partial k}{\partial x_{j}}\right]+\rho \left(P_{k}-\varepsilon \right),$$

(13b) $$\frac{\partial (\rho \varepsilon )}{\partial t}+\frac{\partial (\rho \varepsilon u_{j})}{\partial x_{j}}=\frac{\partial }{\partial x_{j}}\left[\left(\mu +\frac{\mu_{t}}{\sigma_{\varepsilon }}\right)\frac{\partial \varepsilon }{\partial x_{j}}\right]+\rho \frac{\varepsilon }{k}\left(C_{1}P_{k}-C_{2}\varepsilon \right),$$

where *ε* denotes the dissipation rate of turbulent kinetic energy, and *k* denotes the fluid turbulent kinetic energy. Notice that the known constants used in this model are chosen as $\sigma_{k}=1.0$, $\sigma_{\varepsilon }=1.3$, $C_{1}=1.44$ and $C_{2}=1.92$. The production term of turbulent kinetic energy $P_{k}$ and the turbulent viscosity $\mu_{t}$ are denoted as

(14) $$P_{k}=\frac{\mu_{t}}{\rho }\left(\frac{\partial u_{i}}{\partial x_{j}}+\frac{\partial u_{j}}{\partial x_{i}}\right)\frac{\partial u_{i}}{\partial x_{j}},$$

(15) $$\mu_{t}=\rho C_{\mu }\frac{k^{2}}{\varepsilon },$$

where $C_{\mu }$ is 0.09, and *ε*, *k* are expressed as

(16) $$\varepsilon =\frac{\mu }{\rho }\bar{\left(\frac{\partial u_{i}^{\prime }}{\partial x_{j}}\right)\cdot \left(\frac{\partial u_{i}^{\prime }}{\partial x_{j}}\right)},$$

(17) $$k=\frac{\bar{u_{i}^{\prime }u_{i}^{\prime }}}{2}.$$

The model is solved as a steady state problem and all of the walls are set as no-slip boundaries. The standard wall function method is used for the near wall treatment, which is suitable for the high Reynolds number conditions. In addition, the temperature is assumed constant since the ground test is done under a temperature controllable lab environment. For each case, the simulation runs on a computer with a Pentium Intel Quad Core processor (2.6 GHz) and 8.0 GB of memory. The numerical calculation is finished in the case that the residuals are less than 10${}^{-4}$ and the variation of the drag coefficient is reasonably convergent.

### 3.2. Simulation Results and Analysis

**Figure 8:**
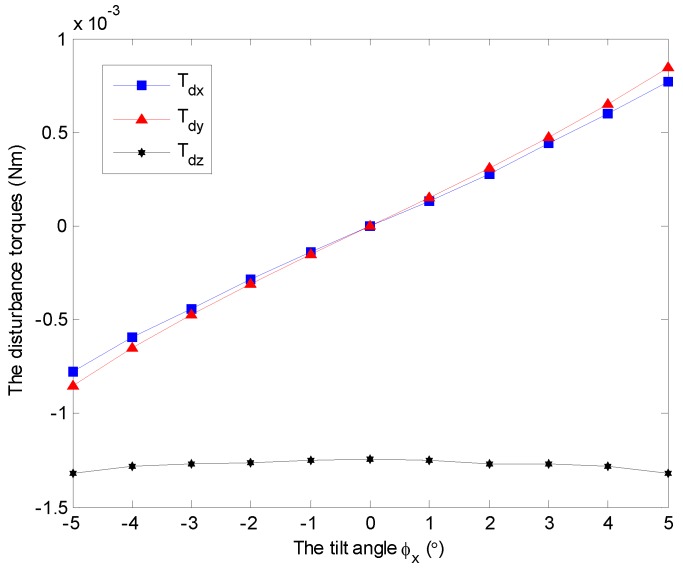
Simulation results of the disturbance torques $T_{dx}$, $T_{dy}$ and $T_{dz}$ with varying tilt angles $\phi_{x}$ under $\phi_{y}=0^{\circ }$ and 3500 rpm.

**Figure 9:**
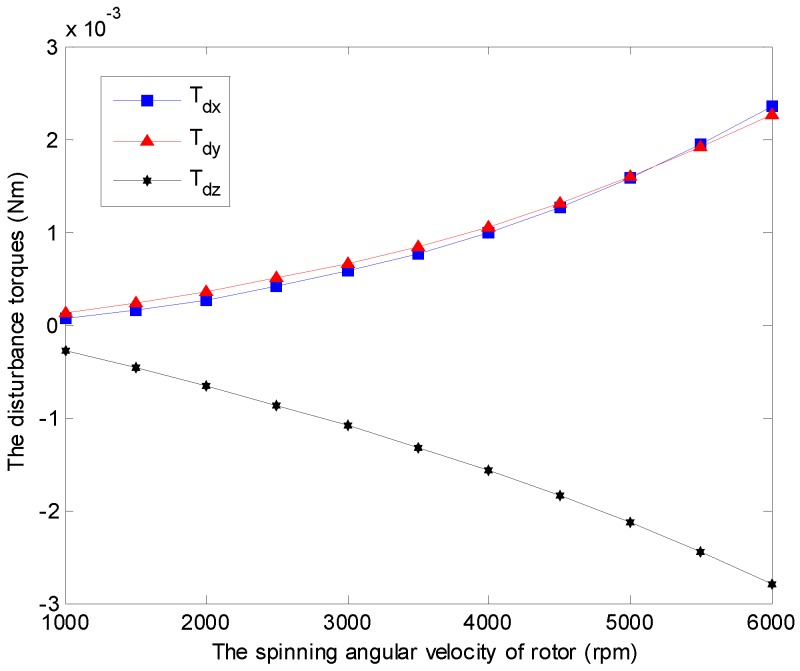
Simulation results of the disturbance torques $T_{dx}$, $T_{dy}$ and $T_{dz}$ with varying angular velocities under $\phi_{x}=5^{\circ }$ and $\phi_{y}=0^{\circ }$.

**Figure 10:**
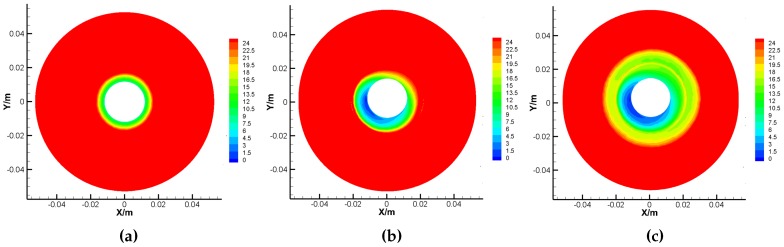
Pressure contours of the upper surface of the rotor under $\phi_{y}=$ 0${}^{\circ }$. (**a**) Results under $\phi_{x}=$ 0${}^{\circ }$ and 3500 rpm; (**b**) Results under $\phi_{x}=$ 5${}^{\circ }$ and 3500 rpm; (**c**) Results under $\phi_{x}=-5^{\circ }$ and 2000 rpm.

**Figure 11:**
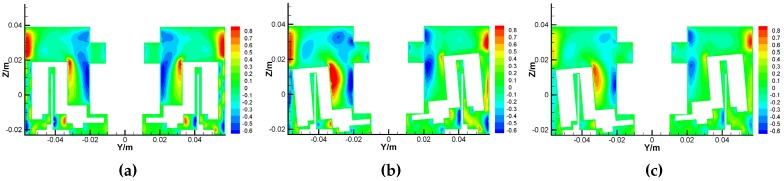
Axial velocity contours of the GyroWheel under $\phi_{y}=$ 0${}^{\circ }$. (**a**) Results under $\phi_{x}=$ 0${}^{\circ }$ and 3500 rpm; (**b**) Results under $\phi_{x}=$ 5${}^{\circ }$ and 3500 rpm; (**c**) Results under $\phi_{x}=5^{\circ }$ and 2000 rpm.

**Figure 12:**
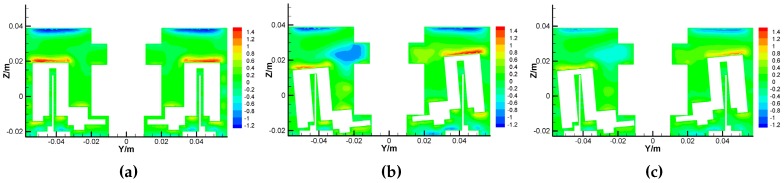
Radial velocity contours of the GyroWheel under $\phi_{y}=$ 0${}^{\circ }$. (**a**) Results under $\phi_{x}=$ 0${}^{\circ }$ and 3500 rpm; (**b**) Results under $\phi_{x}=$ 5${}^{\circ }$ and 3500 rpm; (**c**) Results under $\phi_{x}=5^{\circ }$ and 2000 rpm.

In this section, the main results of this paper are presented, and the aerodynamic drag property is investigated by numerical simulations with the model established in FLUENT. Different cases are considered by changing the spinning angular velocity and tilt angle of the GyroWheel rotor. Without loss of generality and for the sake of simplicity, we assume that the tilt of the rotor is along the *x*${}_{c}$-axis with $\phi_{y}=0^{\circ }$ in the case frame F${}_{c}$. The simulation results are shown in Figure 8, Figure 9, Figure 10, Figure 11 and Figure 12.

Without loss of generality, a constant angular velocity of 3500 rpm is considered here as an example. The aerodynamic disturbance torque with respect to the tilt angle is analyzed, as shown in Figure 8. It follows that the spin disturbance torque $T_{dz}$ remains almost constant, and it hinders the rotation of the rotor. However, the transverse disturbance torques $T_{dx}$ and $T_{dy}$ exist under non-zero tilt angle, and increase with respect to the increasing of tilt angle. Furthermore, the two disturbance torques occur at the same time while the rotor tilts along one axis, which can be observed in both Figure 8 and Figure 9. Similar conclusions can be obtained under other spinning angular velocity conditions, which are ignored here.

The time-varying feature of the spinning angular velocity is an iconic property for the GyroWheel system. In the sequel, the aerodynamic disturbance torque is considered with respect to the variation of the spinning velocity. Figure 9 indicates the relationship between the aerodynamic drag and the spinning angular velocity of GyroWheel rotor where the tilt angle $\phi_{x}=5^{\circ }$ and $\phi_{y}=0^{\circ }$. According to the simulation results, $T_{dx}$, $T_{dy}$ and $T_{dz}$ increase with respect to the spinning angular velocity nonlinearly.

Figure 10 shows the different pressure contours of the upper surface of the rotor under different tilt angle $\phi_{x}$ and spinning angular velocity with $\phi_{y}=0^{\circ }$, where the unit of measure is Pa. Comparing Figure 10a,b, the pressure center is deflected due to the non-uniform gap between the rotor and the case as well as the influence of the centrifugal force acting upon the gas molecules, while the GyroWheel rotor is operated under tilting condition. This deflection of pressure center leads to both of the transverse disturbance torques $T_{dx}$ and $T_{dy}$. Comparing Figure 10b,c, where value is bigger with the increasingly deepened red, the pressure around the rotor increases with respect to the spinning angular velocity.

The airflow motions around the GyroWheel rotor mainly consist of tangential movement and secondary flow, and the latter is formed by the axial motion and radial motion as shown in Figure 11 and Figure 12, where the unit of measure is m/s. Clearly, the secondary flow reaches its maximum near the wall where the red and blue regions mean the opposite flow directions. Comparing Figure 11a,b and Figure 12a,b, the tilt angle leads to the asymmetry of the secondary flow, which can influence the operation of the GyroWheel rotor. Comparing Figure 11b,c and Figure 12b,c, the deep color regions decrease while the spinning angular velocity is reduced. This means that the intensity of the secondary flow is reduced. In fact, the asymmetry of the secondary flow also affects the transverse disturbance torques $T_{dx}$ and $T_{dy}$ to some extent. As the main movement, the tangential velocity leads to the frictional resistance that opposes rotation and dissipates the energy of the system due to the viscosity of air.

Above all, the aerodynamic drag is a significant term for the GyroWheel system in the ground test condition. The non-zero tilt angle leads to the change of the pressure distribution and the asymmetry of the secondary flow, which cause the transverse disturbance torques $T_{dx}$ and $T_{dy}$. Furthermore, the transverse disturbance torques increase with respect to both the tilt angle and the spinning angular velocity of the GyroWheel rotor. In addition, the spin disturbance torques $T_{dz}$, mainly arising from the frictional resistance, are hardly affected by the tilt angle, but increase with the spinning angular velocity directly.

## 4. Experimental Verifications

**Figure 13:**
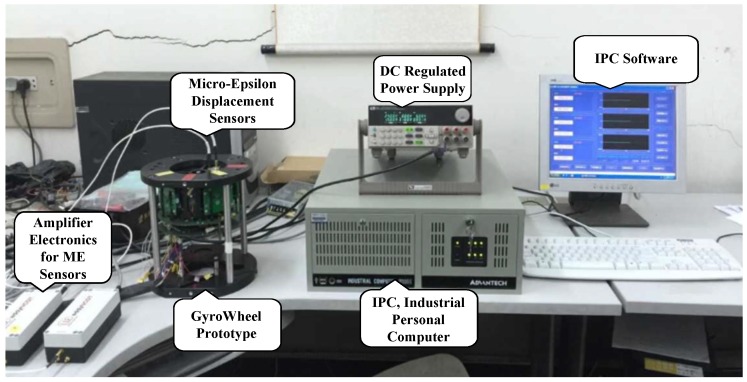
Experimental platform of the GyroWheel system.

In order to demonstrate the practicability and validity of numerical simulation results in Section 3, an experimental platform is set up based on the GyroWheel prototype, as shown in Figure 13. It should be noted that we use an IPC-based system to achieve the control algorithm and logical operation of the GyroWheel system in this step, where the operating system is Microsoft Windows nested with a real-time subsystem (RTSS). The Windows platform provides Human Machine Interface (HMI), while uniform time sampling, real-time control calculations as well as data transmission are operated in RTSS, with a fixed sampling time of 0.5 ms. Some PCI-based boards are adopted to implement counter and the conversions between analog and digital signals, and the spinning of the GyroWheel rotor is controlled by a set of PLL (Phase-Locked Loop) electronics. The tilt angles of the GyroWheel rotor are measured by two-channel Micro-Epsilon displacement sensors, and the spinning angular velocity is obtained by real-time calculation according to the differential of Hall sensors. The platform is powered by a 28 V DC regulated power supply, and a series of precision resistors are used to measure the voltages of the torque coils.

**Figure 14:**
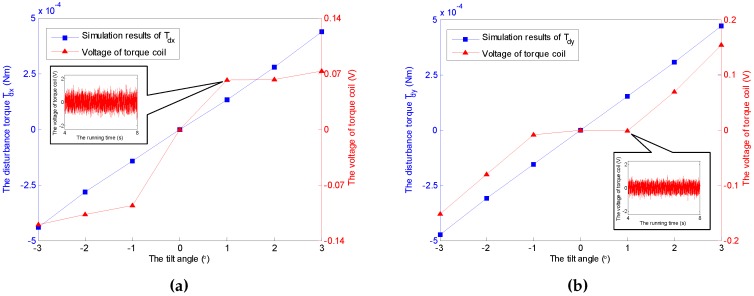
Comparisons of $T_{dx}$ and $T_{dy}$ with varying tilt angles $\phi_{x}$ under $\phi_{y}=0^{\circ }$ and 3500 rpm. (**a**) Comparison of $T_{dx}$; (**b**) Comparison of $T_{dy}$.

**Figure 15:**
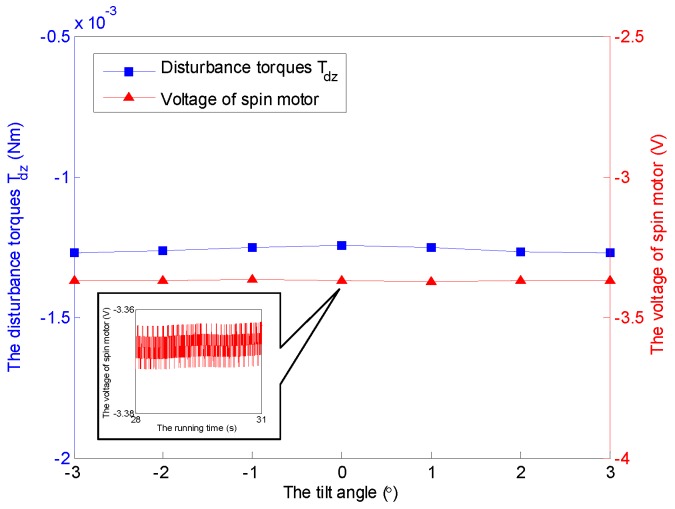
Comparison of $T_{dz}$ with varying tilt angles $\phi_{x}$ under $\phi_{y}=0^{\circ }$ and 3500 rpm.

**Figure 16:**
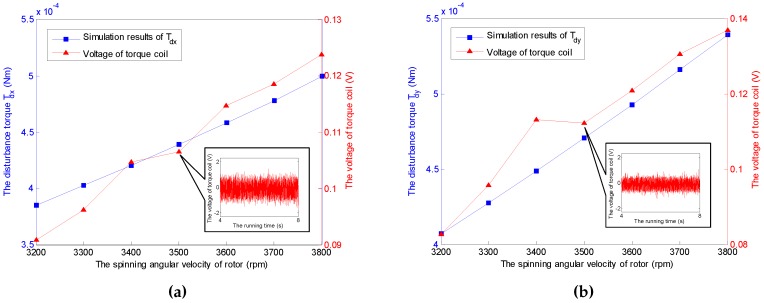
Comparisons of $T_{dx}$ and $T_{dy}$ with varying angular velocities under $\phi_{x}=$ 3${}^{\circ }$ and $\phi_{y}=$ 0${}^{\circ }$. (**a**) Comparison of $T_{dx}$; (**b**) Comparison of $T_{dy}$.

**Figure 17:**
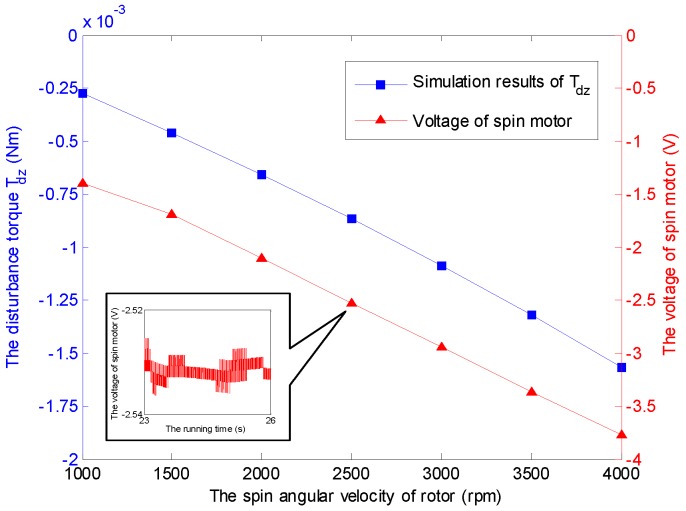
Comparison of $T_{dz}$ with varying angular velocities under $\phi_{x}=$ 3${}^{\circ }$ and $\phi_{y}=$ 0${}^{\circ }$.

In our illustrated experiments, the tilt angle of GyroWheel rotor is limited within ±3${}^{\circ }$ for the sake of security, and the angular velocity varying from static state to a maximum value of 4000 rpm, where we use 1000 rpm as the minimum value for the comparisons. The voltages of torque coils and spin motor, which reflect the corresponding aerodynamic drags in three directions, are sampled. The high-frequency noises, the fundamental frequency mainly caused by rotor unbalance and the second harmonic components caused by gimbal assembly are attenuated by utilizing some notch filters. Finally, the DC components of the actual data are obtained and used for comparision with the simulation results, as shown in Figure 14, Figure 15, Figure 16 and Figure 17.

**Remark** **1.** Due to the kinematics of the GyroWheel system with flex-gimbal suspension, which is similar to DTG in physical configuration, there exist mixed-frequency vibrations, including a variety of multiplier-frequency and beat-frequency signals with respect to the spinning angular velocity (see [8,11,12,13,17] for details and the references therein).

It should be noticed that the obtained voltages in experiments can not be converted to the torques directly due to the unknown moment coefficients of torque coils that are nonlinearly variable with the change of tilt angle, spinning angular velocity, run time and heating. Therefore, the voltage increments of the torque coils and the spin motor are used here to describe the variations of the corresponding transverse disturbance torques and the spin torque, while the tilt angle and the spinning angular velocity vary. According to the simulation cases under a constant spinning angular velocity of 3500 rpm and $\phi_{y}=0^{\circ }$, the relationship between tilt angle of the GyroWheel rotor and the extra consumed voltages are illustrated in Figure 14 and Figure 15, which suggest that the tilting along one axis can lead to two-axis disturbance torques in transverse channels, which increase with respect to the increasing of tilt angle; however, the spin disturbance torque has nothing to do with the change of tilt angle. In Figure 16 and Figure 17, the spinning angular velocity is increased gradually, while $\phi_{x}$ is maintained at $3^{\circ }$ and $\phi_{y}$ is maintained at $0^{\circ }$. The results indicate that the aerodynamic disturbance torques, both of the transverse disturbance torques $T_{dx}$, $T_{dy}$ and the spin disturbance torque $T_{dz}$ increase with respect to the increasing spinning angular velocity of the GyroWheel rotor, which is also consistent with the numerical simulations.

**Remark** **2.** Considering the uneven mass distribution, manufacturing and installation, the isotropy of the GyroWheel rotor is unrealistic. Additionally, the conditions of the airflow around the prototype rotor, including the temperature, uniformity and tightness, are not ideal. All of these lead to some minor differences between the simulation and experimental results. On the other hand, the anisotropy of the GyroWheel rotor leads to the asymmetry of the experiment results, such as the voltages of torque coils in Figure 14a,b and Figure 16a,b.

## 5. Conclusions

In this paper, the aerodynamic drag problem in ground test is investigated and analyzed based on numerical simulations in FLUENT for a novel ADCS device, called GyroWheel, which combines an actuator with rate sensing capabilities. In addition, some experimental studies are developed to verify the correctness of the numerical illustrations. The aerodynamic drag includes two orthogonal transverse disturbance torques and one spin disturbance torque, both of which are damping torques. The spin disturbance torque increases with respect to the spinning angular velocity of the GyroWheel rotor; however, it is hardly influenced by the tilt angle of the rotor. Compared with this, the non-zero tilt angle will cause the transverse disturbance torques, which increase with respect to both the tilt angle and spinning angular velocity of the GyroWheel rotor due to the variation of the pressure distribution and the asymmetry of the secondary flow.

Above all, the aerodynamic drag is a source of interference for the ground test of GyroWheel system. Hence, insight into the characteristics of aerodynamic drag can help to understand the behaviours of the GyroWheel system and propose a better, more effective and feasible ground test scheme without a vacuum tank that will, in turn, reduce the cost of testing. In addition, it is very valuable and necessary to study the precision servo control problem of the GyroWheel system considering the aerodynamic drag, which, in turn, improves the stability and accuracy specification of the system. Future research will focus on these topics.

## Figures and Tables

**Table 1:** Physical parameters of the GyroWheel rotor.

Parameter	Value
Tilt angle of spin axis, *ϕ*	±5${}^{\circ }$
Spinning angular velocity, ${\dot{\theta }}_{z}$	3500 ± 500 rpm
Moment of inertia along the $x_{r}$ axis, $I_{rx}$	1.062 × $10^{-3}$ kg·m${}^{2}$
Moment of inertia along the $y_{r}$ axis, $I_{ry}$	1.062 × $10^{-3}$ kg·m${}^{2}$
Moment of inertia along the $z_{r}$ axis, $I_{rz}$	1.779 × $10^{-3}$ kg·m${}^{2}$

**Table 2:** The simulation parameters.

Structure Parameter	Value
Tilt angle along the $x_{c}$ axis, $\phi_{x}$	±5${}^{\circ }$
Tilt angle along the $y_{c}$ axis, $\phi_{y}$	0${}^{\circ }$
Radius of GyroWheel rotor, $R_{r}$	53.5 mm
Height of GyroWheel rotor, $H_{r}$	38 mm
Radius of GyroWheel case, $R_{c}$	57.5 mm
Height of GyroWheel case, $H_{c}$	62 mm
Temperature	20 ${}^{\circ }$C
Atmospheric pressure, $p_{0}$	101,325 Pa
Density of air, *ρ*	1.205 kg/m${}^{3}$
Coefficients of viscosity of air, *μ*	1.81 × 10${}^{-5}$ Pa·s

## Abbreviations

The following abbreviations are used in this manuscript:

DOF	Degree of Freedom
IPC	Industrial Personal Computer
CFD	Computational Fluid Dynamics
DC	Direct Current
DSP	Digital Signal Processor
PCI	Peripheral Component Interconnect

## Appendix A. Lagrange Equations of the GyroWheel System

The potential and kinetic energy expressions for the GyroWheel system are illustrated as follows, where $\theta_{x}$, $\theta_{y}$, $\theta_{z}$ are chosen to be generalized coordinates:

$$V=K_{x}\theta_{x}^{2}+K_{y}\theta_{y}^{2},$$

$$T=\frac{1}{2}\left[{\omega_{m}}^{T}I_{m}\omega_{m}+{\omega_{g}}^{T}I_{g}\omega_{g}+{\omega_{r}}^{T}I_{r}\omega_{r}\right],$$

where $I_{m}$, $I_{g}$ and $I_{r}$ are the moment of inertias of the motor shaft, gimbal and rotor,

$$I_{m}=\left[\begin{array}{ccc} 0 & \;0 & \;0\\ 0 & \;0 & \;0\\ 0 & \;0 & \;I_{mz} \end{array}\right]\;I_{g,r}=\left[\begin{array}{ccc} I_{g,rx} & \;0 & \;0\\ 0 & \;I_{g,ry} & \;0\\ 0 & \;0 & \;I_{g,rz} \end{array}\right].$$

Therefore, the Lagrange function *L* is obtained as follows:

$$L=T-V=\frac{1}{2}\left[{\omega_{m}}^{T}I_{m}\omega_{m}+{\omega_{g}}^{T}I_{g}\omega_{g}+{\omega_{r}}^{T}I_{r}\omega_{r}\right]-\left(K_{x}\theta_{x}^{2}+K_{y}\theta_{y}^{2}\right).$$

Considering the control torque provided by the torque coils, the aerodynamic drag and the damping torques of torsion elements, the Lagrange equations of the GyroWheel system can be expressed as follows:

$$\left[\begin{array}{c} \frac{d}{dt}\left(\frac{\partial L}{\partial {\dot{\theta }}_{x}}\right)\\ \frac{d}{dt}\left(\frac{\partial L}{\partial {\dot{\theta }}_{y}}\right)\\ \frac{d}{dt}\left(\frac{\partial L}{\partial {\dot{\theta }}_{z}}\right) \end{array}\right]-\left[\begin{array}{c} \frac{\partial L}{\partial \theta_{x}}\\ \frac{\partial L}{\partial \theta_{y}}\\ \frac{\partial L}{\partial \theta_{z}} \end{array}\right]=\left[\begin{array}{c} T_{gx}-C_{x}{\dot{\theta }}_{x}\\ T_{gy}-C_{y}{\dot{\theta }}_{y}\\ T_{gz} \end{array}\right]-\left[\begin{array}{ccc} C_{\theta_{y}} & \;0 & \;S_{\theta_{y}}\\ 0 & \;1 & \;0\\ -C_{\theta_{x}}S_{\theta_{y}} & \;-S_{\theta_{x}} & \;C_{\theta_{x}}C_{\theta_{y}} \end{array}\right]\left[\begin{array}{c} T_{dx}\\ T_{dy}\\ T_{dz} \end{array}\right].$$

Substituting the Lagrange function *L* into the Lagrange equations of the GyroWheel system, the dynamical model of GyroWheel system (see (6)) can be obtained, where $I_{m1}$, $I_{m2}$, $I_{ms}$ are as follows:

$$\left\{\begin{array}{c} I_{m1}=I_{gx}+I_{rx}C_{\theta_{y}}^{2}+I_{rz}S_{\theta_{y}}^{2},\\ I_{m2}=\frac{1}{2}\left(I_{rz}-I_{rx}\right)C_{\theta_{x}}S_{2\theta_{y}},\\ I_{m3}=I_{mz}+\left(I_{rx}S_{\theta_{y}}^{2}+I_{rz}C_{\theta_{y}}^{2}+I_{gz}\right)C_{\theta_{x}}^{2}+\left(I_{ry}+I_{gy}\right)S_{\theta_{x}}^{2}. \end{array}\right.$$

## Appendix B. Nonlinear Torque Term

The concrete expression of the nonlinear torque term $F_{nl}$ in (6) is shown as follows:

$$\left\{\begin{array}{cc} F_{nl1}= & -\left[\left(I_{rz}-I_{rx}\right)C_{2\theta_{y}}-I_{ry}\right]C_{\theta_{x}}\cdot {\dot{\theta }}_{z}{\dot{\theta }}_{y}-\left(I_{gz}-I_{gy}-I_{ry}+I_{rx}S_{\theta_{y}}^{2}+I_{rz}C_{\theta_{y}}^{2}\right)\frac{S_{2\theta_{x}}}{2}{\dot{\theta }}_{z}^{2}\\ & -\left(I_{rz}-I_{rx}\right)S_{2\theta_{y}}\cdot {\dot{\theta }}_{x}{\dot{\theta }}_{y},\\ F_{nl2}= & \left(I_{rz}-I_{rx}\right)\frac{S_{2\theta_{y}}}{2}\cdot {\dot{\theta }}_{x}^{2}+\left[\left(I_{rz}-I_{rx}\right)C_{2\theta_{y}}-I_{ry}\right]C_{\theta_{x}}\cdot {\dot{\theta }}_{z}{\dot{\theta }}_{x}-\left(I_{rz}-I_{rx}\right)C_{\theta_{x}}^{2}\frac{S_{2\theta_{y}}}{2}{\dot{\theta }}_{z}^{2},\\ F_{nl3}= & -\left(I_{gy}+I_{ry}-I_{gz}-I_{rz}C_{\theta_{y}}^{2}-I_{rx}S_{\theta_{y}}^{2}\right)S_{2\theta_{x}}\cdot {\dot{\theta }}_{x}{\dot{\theta }}_{z}-\left[\left(I_{rz}-I_{rx}\right)C_{2\theta_{y}}+I_{ry}\right]C_{\theta_{x}}\cdot {\dot{\theta }}_{x}{\dot{\theta }}_{y}\\ & +\left(I_{rz}-I_{rx}\right)S_{\theta_{x}}S_{\theta_{y}}C_{\theta_{y}}\cdot {\dot{\theta }}_{x}^{2}+\left(I_{rz}-I_{rx}\right)C_{\theta_{x}}^{2}S_{2\theta_{y}}\cdot {\dot{\theta }}_{y}{\dot{\theta }}_{z}. \end{array}\right.$$
